# The complete chloroplast genome sequence of *Aristolochia manshuriensis* Kom. (Aristolochiaceae)

**DOI:** 10.1080/23802359.2019.1675484

**Published:** 2019-10-11

**Authors:** Kyeonghee Kim, Chae Eun Lim

**Affiliations:** Plant Resources Division, National Institute of Biological Resources, Incheon, The Republic of Korea

**Keywords:** *Aristolochia manshuriensis*, medicinal plant, chloroplast genome, Aristolochiaceae

## Abstract

*Aristolochia manshuriensis* is a medicinal plant belonging to the family Aristolichiaceae. In this study, complete chloroplast (cp) genome sequence of *A. manshuriensis* was characterized through *de novo* assembly with next-generation sequencing data. The cp genome is 160,182 bp long and has a typical quadripartite organization consisting of a large single-copy (LSC), a small single-copy (SSC), and a pair of inverted repeats (IRs). The cp genome harboured 79 protein-coding genes, 30 tRNA genes, and 4 rRNA genes. Phylogenetic analysis revealed that *A. manshuriensis* has close relationship with *Aristolochia macrophylla*.

The genus *Aristolochia* L. sensu lato (Aristolochiaceae), which consists of ∼400 species, is distributed from temperate to tropical regions around the world (Ohi-Toma et al. 2006). In Korea, only two species are distributed; *Aristolochia contorta* and *Aristolochia manshuriensis* (Oh 2007). For thousands of years, *Aristolochia* species have been used as medicinal plants in East Asia including China, Korea, and Japan (Chinese Company of Medicinal Materials 1995; Hu et al. 2004). In particular, aristolochic acid (AA) from root, rootstock or stem of *Aristolochia* species is used to treat gout, rheumatoid arthritis, wound festering, or to reduce inflammation (Negi et al. 2003; Heinrich et al. 2009; Nie et al. 2015). However, AA is also reported as mutagenic, nephrotoxic, and carcinogenic to animals and humans (Arlt et al. 2002, 2007; International Agency for Research on Cancer 2002; Cheng et al. 2006; Huang et al. 2007; Nie et al. 2015). Recently, it has been banned to utilize AA for medicinal purposes in many countries (International Agency for Research on Cancer 2002; Cheng et al. 2006; Arlt et al. 2007; Martena et al. 2007; Lai et al. 2010).

In this study, we determined the chloroplast (cp) genome of *A. manshuriensis* to contribute to the classification and development of DNA markers for the authentication of *Aristolochia* species. The specimen was collected from Samil-ri, Sanae-myeon, Hwacheon-gun, Gangwon-do, South Korea (38°0′23.5″N, 127°31′22.2″E) and deposited at NIBR herbarium (KB) with the accession number NIBR-VP0000575956. Total genomic DNA was prepared and sequenced by the Illumina MiSeq platform (Illumina Inc., San Diego, CA) and obtained high-quality paired-end reads of ca. 2.5 Gb. The complete cp genome of *A. manshuriensis* was revealed to GenBank (Accession no. MN132862), as described previously (Kim et al. 2015).

The cp genome was 160,182 bp in length with 38.7% overall GC content. The cp genome structure of *A. manshuriensis* has the typical quadripartite organization featuring two copies (IRa and IRb) of inverted repeat (IR) regions (25,691 bp) that are separated by a large single-copy (LSC) region (89,503 bp), and a small single-copy (SSC) region (19,297 bp). The total number of identified encoded genes is 113 with 79 protein-coding genes, 30 tRNA genes, and 4 rRNA genes.

To understand the phylogenetic relationship of *A. manshuriensis* with relative taxa, a maximum-likelihood (ML) tree was constructed using 75 common protein-coding genes of *A. manshuriensis* and 11 taxa in Aristolochiaceae. Outgroup includes two species of *Piper* (*Piper autrittum* and *Piper kadsura*) in Piperaceae, which is the most likely sister group of Aristolochiaceae (Figure 1). The *Aristolochia* clade comprised well-supported monophyletic group (pp = 100), and the two major subclades were circumscribed; these include (1) subgn. *Aristolochia*, and (2) subgn. *Siphisia* (Murata et al. 2001) (Figure 1). *Aristolochia manshuriensis* was contained in subgn. *Siphisia* clade with *Aristolochia kaempferi*, *Aristolochia kunmingensis*, *Aristolochia macrophylla*, *Aristolochia mollissima*, and *Aristolochia moupinensis*. Among those species, *A. manshuriensis* has more close relationship with *A. macrophylla* (Figure 1).

**Figure 1.**
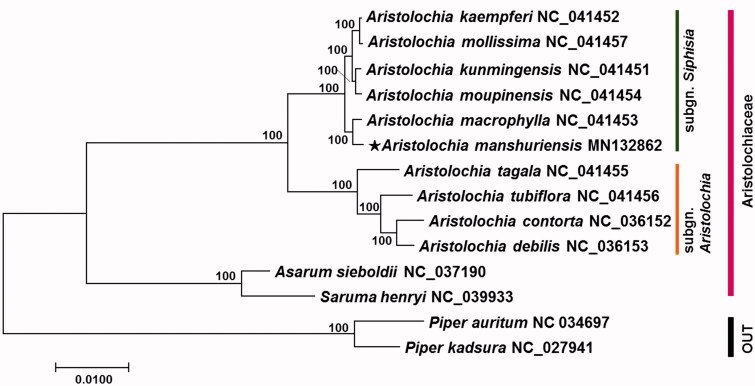
Maximum-likelihood (ML) tree based on the 75 chloroplast protein-coding genes of 14 taxa including *A. manshuriensis*. Sequences of 75 chloroplast protein-coding gene from 14 taxa were aligned using MAFFT (http://mafft.cbrc.jp/alignment/server/index.html) and used to generate ML phylogenetic tree by MEGA 7.0 (Kumar et al. 2016).

## References

[CIT0001] Arlt VM, Stiborová M, Schmeiser HH 2002 Aristolochic acid as a probable human cancer hazard in herbal remedies: a review. Mutagenesis. 17(4):265–277. 12110620 10.1093/mutage/17.4.265

[CIT0002] Arlt VM, Stiborová M, Vom Brocke J, Simões ML, Lord GM, Nortier JL, Hollstein M, Phillips DH, Schmeiser HH 2007 Aristolochic acid mutagenesis: molecular clues to the aetiology of Balkan endemic nephropathy-associated urothelial cancer. Carcinogenesis. 28(11):2253–2261. 17434925 10.1093/carcin/bgm082

[CIT0003] Cheng CL, Chen KJ, Shih PH, Lu LY, Hung CF, Lin WC, Yesong GJ 2006 Chronic renal failure rats are highly sensitive to aristolochic acids, which are nephrotoxic and carcinogenic agents. Cancer Lett. 232(2):236–242. 16458120 10.1016/j.canlet.2005.02.021

[CIT0004] Chinese Company of Medicinal Materials. 1995 China medicinal herbs most in use. Beijing (China): Science Publishing House; p. 838–839.

[CIT0005] Heinrich M, Chan J, Wanke S, Neinhuis C, Simmonds M 2009 Local uses of *Aristolochia* species and content of nephrotoxic aristolochic acid 1 and 2–A global assessment based on bibliographic sources. J Ethnopharmacol. 125(1):108–144. 19505558 10.1016/j.jep.2009.05.028

[CIT0006] Hu SL, Zhang HQ, Chan K, Mei QX 2004 Studies on the toxicity of *Aristolochia manshuriensis* (Guanmuton). Toxicology. 198(1–3):195–201. 15138042 10.1016/j.tox.2004.01.026

[CIT0007] Huang CC, Chen PC, Huang CW, Yu J 2007 Aristolochic acid induces heart failure in zebrafish embryos that is mediated by inflammation. Toxicol Sci. 100(2):486–494. 17823451 10.1093/toxsci/kfm235

[CIT0008] International Agency for Research on Cancer 2002 Some traditional herbal medicines, some mycotoxins, naphthalene and styrene. IARC Monogr Eval Carcinog Risks Hum. 82:1–556. 12687954 PMC4781602

[CIT0009] Kim K, Lee SC, Lee J, Yu Y, Yang K, Choi BS, Koh HJ, Waminal NE, Choi HI, Kim NH, et al. 2015 Complete chloroplast and ribosomal sequences for 30 accessions elucidate evolution of *Oryza* AA genome species. Sci Rep. 5(1):15655. 26506948 10.1038/srep15655 PMC4623524

[CIT0010] Kumar S, Stecher G, Tamura K 2016 MEGA7: Molecular Evolutionary Genetics Analysis version 7.0 for bigger datasets. Mol Biol Evol. 33(7):1870–1874. 27004904 10.1093/molbev/msw054 PMC8210823

[CIT0011] Lai MN, Wang SM, Chen PC, Chen YY, Wang JD 2010 Population-based case-control study of Chinese herbal products containing aristolochic acid and urinary tract cancer risk. J Natl Cancer Inst. 102(3):179–186. 20026811 10.1093/jnci/djp467 PMC2815723

[CIT0012] Martena MJ, van der Wielen JCA, van de Laak LFJ, Konings EJM, de Groot HN, Rietjens I 2007 Enforcement of the ban on aristolochic acids in Chinese traditional herbal preparations on the Dutch market. Anal Bioanal Chem. 389(1):263–275. 17486320 10.1007/s00216-007-1310-3

[CIT0013] Murata J, Ohi T, Wu S, Darnaedi D, Sugawara T, Nakanishi T, Murata H 2001 Molecular phylogeny of *Aristolochia* (Aristolochiaceae) inferred from *matK* sequences. Acta Phytotax Geobot. 52(1):75–83.

[CIT0014] Negi PS, Anandharamakrishnan C, Jayaprakasha GK 2003 Antibacterial activity of *Aristolochia bracteata* root extracts. J Med Food. 6(4):401–403. 14977452 10.1089/109662003772519994

[CIT0015] Nie W, Lv Y, Yan L, Chen X, Lv H 2015 Prediction and characterization of the system effects of aristolochic acid: a novel joint network analysis towards therapeutic and toxicological mechanisms. PLoS One. 5:17646. 10.1038/srep17646 PMC4664954 26620132

[CIT0016] Oh BU 2007 *Aristolochia* In: Park CW, editor. The genera of vascular plants of Korea. Seoul (Korea): Academy Publishing Company; p. 153–154.

[CIT0017] Ohi-Toma T, Sugawara T, Murata H, Wanke S, Neinhuis C, Murata J 2006 Molecular phylogeny of *Aristolochia* sensu lato (Aristolochiaceae) based on sequences of *rbcL*, *matK*, and *phyA* genes, with special reference to differentiation of chromosome number. Synth Bot. 31(3):481–492.

